# "Done more for me in a fortnight than anybody done in all me life." How welfare rights advice can help people with cancer

**DOI:** 10.1186/1472-6963-10-259

**Published:** 2010-09-03

**Authors:** Suzanne Moffatt, Emma Noble, Catherine Exley

**Affiliations:** 1 Institute of Health & Society, Faculty of Medical Sciences, Baddiley-Clarke Building, Newcastle University, Richardson Road, Newcastle upon Tyne, NE2 4AX, UK

## Abstract

**Background:**

In the UK many people with cancer and their carers do not have easy access to the welfare benefits to which they are entitled adding further strain to the process of dealing with cancer. It is estimated that nine out of ten cancer patients' households experience loss of income as a direct result of cancer, which, due to its socio-economic patterning disproportionately affects those most likely to be financially disadvantaged. In the UK proactive welfare rights advice services accessed via health care settings significantly increase benefit entitlement among people with health problems and this paper reports on a qualitative study examining the impact of a welfare rights advice service specifically designed for people affected by cancer and their carers in County Durham, North East England (UK).

**Methods:**

Twenty two men and women with cancer or caring for someone with cancer who were recipients of welfare rights advice aged between 35 and 83 were recruited from a variety of health care and community settings. Semi-structured interviews were undertaken and analysed using the Framework method.

**Results:**

Most of the participants experienced financial strain following their cancer diagnosis. Participants accessed the welfare rights service in a variety of ways, but mainly through referral by other professionals. The additional income generated by successful benefit claims was used in a number of ways and included offsetting additional costs associated with cancer and lessening the impact of loss of earnings. Overall, receiving welfare rights advice eased feelings of stress over financial issues at a time when participants were concerned about dealing with the impact of cancer. Lack of knowledge about benefit entitlements was the main barrier to accessing benefits, and this outweighed attitudinal factors such as stigma and concerns about benefit fraud.

**Conclusions:**

Financial strain resulting from a cancer diagnosis is compounded in the UK by lack of easy access to information about benefit entitlements and assistance to claim. Proactive welfare rights advice services, working closely with health and social care professionals can assist with the practical demands that arise from dealing with the illness and should be considered an important part of a holistic approach to cancer treatment.

## Background

In the UK, many people with cancer and their carers do not have easy access to the welfare benefits to which they are entitled [1]. This may result in difficulties affording the additional resources required to cope with illness [2], as well as adding further strain to the process of dealing with cancer. However, such financial pressures are not often discussed with health professionals [3,4]. Evidence shows that cancer is likely to affect patients' incomes although there appear to be differential effects depending on the type of cancer [5] and whether those affected are still in employment or retired [6]. Surviving cancer is associated with unemployment [7] and loss of earnings disproportionately affects those in low income groups [8]. Recently, gender differences in income following cancer diagnosis and treatment have been observed. Cancer in men may result in greater financial difficulties than cancer among women (in couple households), but women's earnings were lower after both their own and their spouses' cancer [8].

In most developed countries, welfare states have developed systems to compensate for unemployment as a result of illness, although the level of recompense and the means by which it may be accessed varies [9]. The UK state welfare system has a series of means tested and non-means tested benefits designed to assist those who have ongoing health problems, as well as benefits aimed at those who cannot work (either temporarily or permanently) due to ill-health. However, each year in the UK, billions of pounds of benefits remain unclaimed [1]. Services have been developed to assist citizens to claim their entitlements, although these are often *ad hoc *and short term arrangements. Perhaps the most successful of these are proactive welfare rights advice services accessed via health care settings, which have repeatedly been found to significantly increase benefit entitlement among those with health problems [10]. Within the UK, assisting people to obtain their full welfare benefit entitlement was advocated by the Acheson Report as an intervention with the potential to reduce inequalities in health [11] and has been recognised as an important response to tackling social and health inequalities by the UK's Treasury [12] and the Social Exclusion Unit [13]. Additional resources (whether financial or other material benefits such as aids and adaptations) have been shown to reduce stress, improve individuals' ability to cope with health problems and improve quality of life [14,15]. Many people with long-term health conditions and their carers face considerable financial burdens as a result of their illness. For those of working age, income may drop because of time off work for treatment, job loss or the difficulties associated with re-entering employment [8]. For those over state pension age, the additional costs of dealing with ill-health while on a fixed income can have serious financial implications [16]. In the UK, one in five people over the state pension age live in poverty [17] and there are currently 23 different types of benefit available to older people, many of which are linked in a complex network of entitlements [18]. The National Audit Office has criticised the UK benefits system as unnecessarily complex [18].

In the UK, nine out of ten cancer patients' households experience loss of income as a direct result of cancer [1]. This may be due to temporary or permanent loss of earnings as well as the additional costs associated with cancer such as travel costs to treatment centres. Despite this, among people with cancer and their carers, there is significant under claiming of health-related benefits that has been attributed to a number of factors including: lack of knowledge about benefit entitlements, the complexities of the benefits system and the lack of services designed to facilitate access to benefits. Within the UK health care system it is often unclear who, if anyone, has responsibility or capacity for dealing with the social aspects of illness. The boundaries between medical and social issues are often blurred, and there are no routinely available services with the expertise to deal with sociolegal issues arising from ill-health.

In the UK, the prevalence of most cancers is socio-economically patterned; higher rates of most cancers are found among people from lower occupational classes [19]. There are exceptions to this socio-economic patterning, most notably breast cancer [20,21]. It is the case that in the UK, the prevalence of most cancers are higher within lower socio-economic groups, who are already most likely to be financially disadvantaged. However, it is also the case that those on relatively high incomes pre-diagnosis may experience significant declines in income. Research into the psycho-social aspects of cancer found that patients with fewer social and economic resources had significantly higher levels of need for practical help with matters such as form-filling, transport and housework, and that help with financial matters was identified as a 'significant unmet need' [22] (p601). Additionally, technological advances are leading to increased cure rates and longer survival. These welcome advances can lead to prolonged periods of illness and disability, in some cases leading to remission and in others, death. The cancer trajectory may therefore be long, uncertain and complex and pose difficulties in relation to benefit eligibility criteria, particularly for those of working age.

Within the UK health and social care system, there is therefore a strong case for facilitating access to welfare rights advice for people with cancer and their families in order to assist them to claim their entitlements. This paper reports on interim findings from an evaluation of a dedicated welfare rights advice service for people affected by cancer developed as a result of collaboration between Durham County Council in North East England and Macmillan Cancer Support (a major UK charity that provides practical, medical and financial support and campaigns for better cancer care). We focus on (i) the impact of a cancer diagnosis on finances, (ii) the impact of welfare rights advice, and (iii) issues in accessing welfare rights advice.

### Background to the welfare rights advice service and cancer related benefits

In June 2008 Durham County Council, in collaboration with Macmillan Cancer Support appointed three experienced welfare rights advisors to provide a dedicated service for people with cancer and their carers across County Durham in North East England. County Durham has a population of 504,900 that includes urban, semi-rural and remote rural populations, as well as areas of significant socio-economic deprivation and poor health. Around one third of the population of County Durham live in the most deprived areas of England, with only ten percent living in the least deprived areas [23]. Life expectancy in County Durham for males and females and early deaths from cancer is worse than the average for England [24]. In terms of cancer type, the highest incidence rates in men are for lung, prostate and colorectal cancers; for women the highest incidence rates are for breast, lung and colorectal cancers [25]. The welfare rights advice service was designed to be freely accessible, so that individuals could self refer, as well as be referred by health, social care or voluntary sector professionals. The three staff are based in the local authority offices, but work in various National Health Service (NHS) and voluntary sector locations throughout the county when providing advice. At its inception, the service was widely advertised to the public and professionals and there are ongoing publicity events and talks to maintain the service profile. The welfare rights advisors provide dedicated welfare rights services for people affected by cancer. These include: service delivery in a range of health, social care and voluntary sector settings, home visits, contact by email and telephone. The service comprises a full welfare benefits check followed by assistance to claim entitlements, follow-up work and representation at appeals and tribunals. The welfare rights advisors also undertake outreach work to voluntary and community groups in order to facilitate awareness among the wider public. As well as this, they carry out awareness training for health, social care and voluntary sector staff to increase referral rates and to enable these staff to deliver basic benefit information to increase the reach of the service.

The UK benefits system is designed to provide financial assistance for people with health problems, as well as help for carers, those with children and those on low incomes. Different benefits and rules apply to those above and below state pension age. For those aged 65 and over, Attendance Allowance (AA) can be claimed; those below 65 who require care and attention can claim Disability Living Allowance (DLA) and those who are unable to work can claim Employment Support Allowance (ESA) (formerly Incapacity Benefit). Importantly, AA and DLA are not means tested. Most other benefits are means tested and there is a complex network of benefits that individuals may or may not be entitled to as a result of changing circumstances brought about by ill health. Previous work has highlighted the need for expert knowledge to negotiate the welfare benefits system on behalf of individuals with health problems [15,26]. The UK benefits system has special social security rules to accommodate rapidly progressing terminal cancers where life expectancy is six months or less. In practice, it appears that there is wide variation in doctors' interpretation of the six-month life expectancy definition of terminal illness as well as difficulties in predicting patients' life expectancy [27]. Evidence from people with lung cancer indicates that, despite these procedures, many do not obtain the benefits to which they are entitled because they did not receive timely advice [3].

## Methods

### Research governance

Ethical approval was obtained from Sunderland NHS Local Research Ethics Committee and research governance approval from the Research Management and Governance Unit of County Durham & Tees Valley PCT.

### Sampling and interviews

Between June 2008 and March 2009, 792 individuals were seen by the welfare rights service. During their first welfare rights advice consultation, participants were informed about the research study and completed a baseline questionnaire covering basic demographic information (age, sex, education, employment status, referral route). Participants were given an information sheet about the qualitative study and asked to consider whether they would be willing to take part in an interview about their experiences of the welfare rights service. Those who consented (N = 105) formed the sampling frame for this qualitative interview study.

Purposive sampling was undertaken to achieve maximum variation on the basis of age, sex, type of cancer, index of multiple deprivation (based on postcode) [23] and residential location (urban, semi-rural, remote rural) [28].

A semi-structured topic guide was used that covered: benefits related issues; the impact of cancer on work, family and finances; and, the impact of the welfare rights service. All participants agreed to their interviews being digitally recorded and they were transcribed in full. Interview duration ranged from 22 to 100 minutes. Observational field notes were taken of any factors that influenced the interview, such as level of ill-health or contribution of partner/carer.

As this paper reports on interim findings, we did not seek to reach data saturation in all thematic categories, but we reached saturation in the categories reported. The sample size of 22 is justified on the basis that consistent findings were being made in the key categories that are the topic of this paper, namely: impact of cancer diagnosis on finance; impact of welfare rights advice services; and, accessing welfare rights advice services.

### Analysis

Each of the transcripts were read and re-read by two members of the team (EN and SM) and a conceptual framework was devised. Coding was undertaken by one person (EN) using the coding procedure in Nvivo Version 7 [29], interview elements that were difficult to code were discussed with SM. The data were coded and charted systematically using the Framework method [30] and the resulting typologies discussed by SM, EN and CE. This method of organising the data allows participants' circumstances, experiences and views to be compared within and across groups in a framework derived from their own accounts. Constant comparison [31,32] and deviant case analysis [33] were used for internal validation [34].

## Results

Twenty two interviews were undertaken between January and June 2009, 11 men and 11 women. Seventeen participants were interviewed alone, four people with carers present and one carer was interviewed alone. Participants ranged in age from 35 to 83 and all were white English speakers. Table 1 shows demographic and health information of the study participants. Most participants came from areas with higher levels of deprivation and there were no participants living in areas of lowest deprivation. Participants resided in a range of types of area including urban, semi-rural and rural, although fewest came from the most rural locations. Most participants were not in paid work or unable to work at the time of the interview, one participant was in part time work and one participant had recently returned to work following treatment. Some of those off work due to cancer intended this to be for a relatively short-term period; for others this was long term. Participants experienced a wide range of types of cancer. While we are primarily concerned with reporting the impact of the welfare rights advice service, we contextualise this by describing participants' accounts of the financial impact of a cancer diagnosis, and we conclude the results section with accounts of the existing barriers to claiming benefit entitlements.

**Table 1:** Participants' demographic and health information

Category of information collected		Number of participants
**IMD Quintile **[16]	1 (highest deprivation)	7
	2	8
	3	4
	4	3
	5 (lowest deprivation)	0
		
**Rural/Urban Indicator **[28]	3 (Village-sparse)	1
	5 (Urban-less sparse)	9
	6 (Town & Fringe-less sparse)	7
	7 (Village-less sparse)	5
		
**Employment status**	Back to work	1
	In work part time over 16 hours	1
	Year off work to care	1
	Off work long term (cancer)	4
	Off work short term (cancer)	4
	Off work long term (other morbidity)	3
	Unsure	1
	Retired (over state pension age)	7
		
**Cancer Type**	Bile Duct	1
	Breast	4
	Bowel	2
	Lymphoma	2
	Lung	3
	Melanoma	1
	Myeloid leukaemia	1
	Oesophageal	1
	Parotid gland	1
	Prostate	2
	Spine	1
	Thyroid	1
	Tongue	1
	Not stated	1

### Impact of cancer diagnosis on finance

The severity of the financial impact of a diagnosis of cancer differed depending on the circumstances of the household. For a small group of respondents, the cancer trajectory did not have any financial impact on their lives. This was almost wholly in couple households where a working partner earned a high income and/or the individual was well covered by private health insurance and/or mortgage protection,

*He's a top earner (husband) and we did have insurance ... and that's when we got the money ... and it's been a big, big help ... So financially we're not struggling at all*. (P057, female, aged between 35-39)

The stress of the diagnosis and ensuing treatment was compounded for some people by financial stress. For those of working age, circumstances differed considerably depending on the employer and type of employment. In general, those in better paid jobs were more likely to have health insurance or mortgage protection policies, and those working in the public sector had more generous sickness benefit packages,

*I was fortunate because I work for the [public sector] and we get full pay [for 6 months] and half pay [for 6 months], a lot of people aren't in that position. They come off work and they're just getting sickness benefit ... so you know it does impact on your whole life really you know, financially as well as emotionally and physically*. (P097, female, aged between 45-49, returned to work following cancer treatment)

This position was in stark contrast to those who did not qualify for occupational sickness benefits and who had to rely on Statutory Sick Pay or Employment Support Allowance once cancer was diagnosed and treatment took place,

*It's a devastating thing, you can't work, you can't and it's hard to pay your bills. It's a hard enough worry cancer itself, without having to worry about money as well. That's the major thing with cancer, apart from obviously having the cancer, that's top priority is the financial side*. (P046, male, aged between 35-39, self-employed, stopped work for treatment, having to re-start business)

Participants spoke about the difficulties they encountered when trying to manage on a significantly reduced income prior to receiving their full benefit entitlement. A number of participants appeared to be managing financially only because of the additional finances that resulted from the assistance of the welfare rights advisors,

*I was just on the Statutory Sick Pay ... We were managing because my wife earns quite a lot of money ... she's not too badly off ... You know once you stop earning it's a real blow ... it's a big drop, so it [gaining extra money through Disability Living Allowance] has cushioned it a bit you know*. (P119, male, aged between 55-59)

Most individuals interviewed suffered a considerable drop in their household income and/or significant additional expenditure as a result of their cancer diagnosis, and this was particularly severe for those on low incomes whether below or above state pension age. A number of working age participants had young children at home and several were the 'main' earner (i.e. the earner of the highest salary in the household). For those of working age, the financial impact could be severe as a result of a period of interruption from work or job loss. This was particularly the case for those who were self-employed without illness insurance and those in low paid jobs and jobs without sickness benefits,

*It's hard not getting any money in. My wife had to increase her hours a little bit at work ... which she didn't want to, she wanted to spend more time at home with me so ... it's hard, very hard*. (P046, male, aged between 35-39)

*I think when you're feeling poorly, if you've got money worries it brings you down a little bit further. I think when you haven't got the worry you can sort of concentrate on getting yourself sorted as well as you can*. (P003, female, aged between 70-74)

The shock of a cancer diagnosis and the prospect of the ensuing treatment was participants' foremost concern. Financial worries did not immediately register as participants were too busy dealing with the impact of their diagnosis. Several individuals recounted how they were too shocked and emotionally distressed to consider the need to inform relevant agencies about their changed circumstances and moreover, were reluctant to discuss their health problems with strangers over the telephone. Most participants felt that the period close to diagnosis was not a good time to deal with financial issues. However, for some people, there was an imperative to get to grips with the various benefits related agencies in order to ensure that they did not miss out on benefit entitlements, nor fall foul of the system by continuing to claim for benefits for which their diagnosis disqualified them,

*Obviously it's so upsetting when you get diagnosed with cancer, I didn't even contact the working tax credits and the welfare rights advisor said you have to do this and do that and do the other ... So that was a big help, I hadn't even contacted the people to say I wasn't working anymore, you know, the Job Centre*. (P046, male, aged between 35-39)

Cancer treatment entailed considerable expense for some individuals. Petrol and parking costs for hospital visits, additional heating costs, dietary needs, clothing needs and trying to make ends meet on a lower income were some of the examples cited by participants. A number of people outlined the ways in which they cut back on their expenditure, such as, buying cheaper brands of food, economising on other household bills, reducing social and leisure activities. Some individuals borrowed cash from other family members. The small number of participants with children spoke about trying to maintain a sense of normality and routine, which often meant continuing with activities such as sports and music that usually involved some expense,

*Well, we forked out for a recorder and book, and you don't like to say no because of what's going on around the home as well*. (P090, female, aged between 35-39)

### Impact of welfare rights advice services (i) Process of obtaining advice

The welfare rights advice service operated through two main processes that facilitated access to benefit entitlements, firstly, providing knowledge about entitlement and secondly, assisting with the procedure of making benefit claims. Once benefit entitlement was established, practical assistance to claim was key, particularly form filling and ongoing advice. Those with cancer spoke about a number of physical and emotional problems resulting from their conditions and treatment (pain, tiredness, difficulty concentrating, anxiety, depression) which made it unlikely that, without assistance, they would be able to complete complex forms and deal with the ensuing bureaucracy,

*Let's face it, if you're recovering from cancer, money isn't exactly the main thing you worry about ... I mean it took me four or five months to even think straight*. (P021, male, aged between 70-74)

*It would have been a nightmare to be honest ... finding out what I could have claimed and where, who to ask what, you know, em plus I mean, the form filling. The form filling was the other thing. The welfare rights advisor knew exactly how to fill the forms in ... you don't want to be doing that when you're not very well, do you? *(P119, male, aged between 55-59)

Carers mentioned the degree of physical and emotional strain that they were under that made it difficult or impossible to pursue claims without assistance,

*... because you do worry about the money side of things and um the forms are just horrendous. And you don't know which way to sort of turn, but when you've got somebody there to help and advise you it's brilliant. Because it does take the pressure off you*. (C006, female carer, aged between 50-54)

Although some participants were prepared to complete the application process themselves, they were a minority, and this was a view held only among those who had been diagnosed for some time. However, even among those prepared to apply for benefits themselves, the main barrier to doing so was lack of knowledge about entitlements,

Carer: *If we'd been told about it [benefit entitlement] we would have [applied ourselves] because, you know, we're quite into doing things ourselves*.

Partner: *The surgery [GP] has a mass of leaflets but, er, which we look through occasionally when we're there, but none of them seem to have anything to do with grants, benefits*. (PO15, male, aged between 75-79)

As Table 2 shows, half of the participants had already received one or more benefits as a result of other services, most commonly the Macmillan Cancer Specialist nurses. Most of these benefits were specific to health, often Disability Living Allowance or Attendance Allowance. Despite already having some benefits in place, these participants remained eligible for subsequent benefits, emphasising the need for specialist welfare rights services with expertise in the entire benefits system which is both complex and ever-changing.

**Table 2:** Factors associated with accessing welfare rights advice services

Factors elicited at interview	Number of participants
**Some benefit(s) already in place prior to specialist service**	
Yes	11
No	11
**Number of benefits obtained per person**	
0	3
1	10
2	5
3	3
4	1
**How participant found out about welfare rights advice service**	
Advertising	3
Benefits Agency Worker	1
Cancer Information Centre	1
Cancer Specialist Nurse	4
District Nurse	1
Hospital information pack	1
Macmillan Nurse	5
Macmillan Support Worker	1
Support Group	2
Unsure	1
Word of mouth	1
Work colleague	1
**Referral to welfare rights service**	
Benefits Agency Worker	1
Cancer Information Centre	1
Cancer Specialist Nurse	1
Family/friend	5
Macmillan Nurse	4
Macmillan Support Worker	1
Self	8
Unsure	1
**Location of welfare rights advice**	
Cancer Information Centre	4
Home	17
Telephone	1
**Approximate time between diagnosis and receipt of advice**	
Same Day	2
2-4 weeks	1
1-6 months	5
6-12 months	2
Over 2 years	4
10+ years	2
Unsure	6

Three respondents did not obtain successful outcomes from the claims made on their behalf. Some individuals did not qualify because of the level of their incomes and assets; others were on relatively low incomes but did not qualify for a number of reasons. Firstly, being unable to work because of cancer treatment, but not having sufficiently high health/care needs to qualify for health related benefits. Secondly in the case where a partner continued to work which precluded them from obtaining means tested benefits. Thirdly, ineligibility resulting from insufficient National Insurance payments (particularly in the case of those in education or training). However, despite not receiving any monetary assistance, the individuals concerned found it helpful to have expert advice and someone to assist them with the process of checking what they might be entitled to,

*I was told initially that there probably wasn't anything that I could get, which is actually how it's turned out. I mean that's not the fault of Macmillan, it's obviously, in my opinion, the fault of the system if you like. I think it's disappointing when you've worked your entire life and sort of in the back of your mind, you think well if I'm ill or if something happens, at least, you know, we get this benefit of some kind and then it's not there, that is pretty damning*. (P156, male, aged between 60-64)

### (ii) Outcome of obtaining welfare rights advice

The income generated as a result of welfare rights advice enabled people to afford necessities and additional items that were required as a result of a diagnosis of cancer. The money was used in a number of ways and included: offsetting additional costs associated with cancer (travel and parking costs, heating bills, dietary needs, clothing needs due to weight loss/gain); lessening the impact of loss of earnings or unemployment; providing a safety net while individuals negotiated with companies over their health insurance claims. While financial stress could not be relieved for all participants, for the small number of individuals who did not qualify for additional income, the fact that they had received expert advice and had all avenues explored gave some reassurance. All individuals valued knowing about the service and having somewhere to turn should their circumstances change,

*But without the help I definitely think it would have been a struggle for everybody. I mean we have good families and all that, but that little bit, the extra money has been a godsend. And as far as I'm concerned has taken a lot of pressure off my illness*. (P106, male, aged between 55-59).

*It takes the pressure off ... you know it takes the worry away. You can concentrate on getting yourself better or making your life as comfortable as you can ... it's one less thing to worry about ... It gets one of your main concerns covered if you like. I just know it would have been bad. It would have just been, you know ... I think the worry would have killed us off, not the cancer*. (P017, female, aged between 65-69)

The range of emotions resulting from cancer diagnosis and treatment was, for some individuals, compounded by worries about finance both in the immediate and longer term.

Having described the impact of welfare rights services, we conclude the results section by examining the factors that impeded individuals' access to welfare rights advice and the ways in which study participants accessed these services.

### Barriers to accessing the benefits system

Lack of knowledge of available benefits was the greatest single barrier. Most participants recounted how they did not know that there were benefits available to them and therefore would not think to claim,

*There's no means of knowing what you're entitled to or what you're not*. (P021, male, aged between 70-74)

*I wouldn't have applied for them [benefits] off my own back because as I say, I didn't know I was entitled to anything*. (P036, female, aged between 45-49)

A number of participants were already in receipt of benefits prior to having a diagnosis of cancer. Despite this, help from the welfare rights advisers was required in order to check their current benefit status and changes in entitlement as a result of cancer. Those already receiving benefits were no more likely to pursue a benefit claim independently than those not, nor were they more likely to be aware of their entitlement to benefit.

It was common knowledge among most of our participants that many millions of pounds of benefits in the UK are unclaimed every year. However, the general publicity about benefits did not appear to alert participants to their entitlements. The lack of available information that people could understand and then act on was highlighted as a major concern. Particularly among those for whom it was some time between diagnosis and receiving benefits advice, there was a view that if professionals did not alert them to benefit entitlement, then people would assume they were not entitled,

*I mean we see all these adverts off the TV about people aren't claiming their benefits, but if you don't know what the benefits is, it's not very helpful*. (C005, female, caring for partner with cancer)

*It shocked me that nobody had ever told me about it ... you think that somebody would have told you about it*. (P081, female, aged between 75-79)

This last comment relates to a point raised by a number of participants concerning the number of professionals that they were in contact with who did not alert them to any information about benefit entitlements. A commonly held view was that if no-one informed them of the possibilities of welfare entitlements, then there would be no entitlement.

In describing their lack of knowledge about their own eligibility for benefits, a number of participants, particularly those over retirement age, discussed at some length attitudinal factors that influenced their propensity to claim (had it been left to them alone). Values of hard work, 'making do' and self reliance were narrated by several participants. Most recounted how they had worked hard all their lives and avoided claiming benefits, despite periods of unemployment. Moreover, many related unpleasant dealings with the 'Social Security' system in the past.

*It's just this stigma attached, oh you're claiming this, but I think now I've got to the point where it does help, even with the shopping, so I would advise anyone, yes do claim if you need it, because it does help towards the financial side ... you just have to swallow your pride ... it's really good they've got things in place like that*. (P057, female, aged between 35-39)

Compounding these views, were beliefs about the extent and severity of illness. Some participants felt that they were not as ill as others they had known or currently knew and therefore not genuinely deserving of health-related benefits, despite in some cases serious illness and prognoses. These feelings were, for some, bound up with the process of accepting and dealing with illness; the receipt of benefits symbolising an inability to cope with the financial aspects of life that had previously been managed prior to becoming ill with cancer,

Carer: *It makes us sound thick, but my husband always said, oh I'm not bad enough*.

Partner: *Well, it's partly my attitude ... I fight all along to try to assume that I can cope with [illness] easily ... when I go along to the surgery or anywhere, hospital, whatever, I look around at all the other people in the waiting area and I feel a bit of a fraud*. (PO15, male, aged between 75-79)

Accounts of 'stigma', 'sponging', 'pride' and 'deserving' were cited by a number of people as barriers to *seeking *benefits. None of the interviewees in this study, however, refused to apply for benefits when this was suggested by the welfare rights advisor. Yet, a number of participants mentioned cases of people they knew who were claiming in the absence of a 'genuine' illness. Participants could not understand how people could get away with this and blamed the individuals falsely claiming, those sanctioning claims, but also the system itself. Thus, they wished to distance themselves from the system, but also from those non-genuine claimants, who they felt brought the whole system into disrepute. Participants appeared to distinguish fairly clearly in their own minds between genuine and non-genuine claims, and invoked a strong sense of unfairness when they thought that people were abusing they system,

*The amount of people I knew who never had any illness and they were just pulling the wool over the doctor's eyes was serious ... I mean they must tell some shocking lies to the doctors ... I don't agree with it, but they were getting away with it ... like I say when I had the [serious illness], a genuine illness ... I never claimed anything all these years because I'd always thought of myself as not having a disability even though I knew I had ... people had been telling us for months, 'You should claim,' and eventually I did go and it was so disheartening when I had a genuine illness and I got turned down*. (P078, male, aged between 50-54)

A number of factors acted as barriers to obtaining benefit entitlements. Lack of knowledge and information about entitlements were the greatest barriers. There were also a number of attitudinal factors that appeared to relate to lifetime experiences, concerns about stigma and scrounging, compounded by personal awareness and campaigns about benefit fraud. However, when assisted to claim, no individuals refused to take up their entitlements. Having outlined barriers, we turn finally, to describe the ways in which participants accessed this welfare rights service.

### Accessing welfare rights advice

As shown in Table 2, participants found out about the service in a number of ways, mostly from a professional including Benefits Agency staff, Macmillan nurses, cancer specialist nurses and district nurses. This indicates the importance of the integration of services for people with cancer, alerting them to services that they can call on when needed. Referral to the service included both self-referral and referral by a professional or voluntary sector staff member. There were therefore a number of routes to accessing this service and some individuals commented on how varied knowledge about welfare rights advice services could be depending on where they happened to be receiving treatment,

*When I was at [named] hospital, I just didn't see anybody. As soon as I was at [different hospital] there seemed to be somebody straight there ... as soon as you walked in, what can we do to help? ... [welfare rights advice] didn't seem to be a standard procedure at [named] hospital*. (P058, male, aged between 50-59)

Most participants were seen in their own homes, and this was particularly suitable for individuals who were unwell and fatigued from treatment (many of whom were having to travel long distances for this).

*Great. You know in your home environment. Em, it's a much better, you know, that she actually saw us here, you know. You're more relaxed and, you know, I had all the paperwork here*. (P119, male, aged between 55-59)

All participants agreed that welfare rights services should be available for people affected by cancer, and that this should not be something that medical staff ought to deal with. Participants were uniform in their views about the characteristics of such services - that they should be proactive, offer practical help with information about entitlements and active assistance to claim. Participants' views about the best time to provide information about the advice or advice itself varied and ranged across: point of diagnosis; at the bedside while recovering from surgery; at the point of discharge; and, after chemotherapy. These opinions broadly reflected individual circumstances as well as medical treatment and participants' recollections of how they had felt during their period of treatment. What came across strongly was that welfare rights services were regarded as an important part of non-medical care for people with cancer; comparisons were drawn with other non-medical services that exist to assess people's readiness to cope with their illnesses on leaving medical care,

*...if you're going home from hospital or somebody was in the hospital doing the care thing, coming to the bed and saying, right you're going home now, just the same as an old person who's assessed when they come home to see if they can boil a kettle ... *(P106, male, aged, between 50-59)

The final row of Table 2 shows participants' estimates of the time between diagnosis and their receipt of welfare rights advice, highlighting great variation. Those who had been eligible, but not received benefits for years or months reflects a reality for many people with cancer; without specialist services, there will be substantial non uptake of benefit entitlement. Those who received advice within a matter of days or months were participants who were recently diagnosed and for whom referral to the new service was operating efficiently. Those accessing the service overcame previously mentioned barriers to claiming their entitlements. A range of factors involved in facilitating access have been identified (Table 2). Health professionals and commissioners need to give these careful consideration in order that high quality services tailored to the needs of people with cancer can be provided.

## Discussion

Participants' accounts capture the stress of cancer and the ways in which financial worries may compound dealing with diagnosis and treatment. Our findings bear out those of a larger study into the psycho-social needs of cancer patients which found that financial needs were most likely to be unmet and that for some of those experiencing financial hardship, 'this aspect of living with cancer was almost worse than the disease itself' [35] (p602). The extent of financial problems varied depending on individual circumstances, but had significant and long-term implications for a number of individuals and their families. Despite the existence of state benefits designed to assist people with health problems, very few individuals knew about what might be available and even fewer knew how to claim. Moreover, the effects of cancer diagnosis and treatment presented a further barrier, and few participants felt well enough (physically and emotionally) to undertake the process of claiming themselves. The welfare rights advice service was valued by participants in a number of ways: alerting people to their entitlements; processing claims; and dealing with the complex bureaucracies that surround benefits. The additional monies obtained compensated to varying degrees for extra expenses related to illness and loss of earnings.

This study adds to the existing qualitative literature about the financial impact of cancer on individuals and their families and is one of the first that has included a range of cancer types, people below and above state pension age and individuals from urban, semi-rural and rural areas. Caution must always be exercised in drawing conclusions from small scale qualitative studies, particularly one with the range of case variability present in this sample [36]. In terms of the sampling, the study does not include individuals from ethnic minority groups or people whose first language is not English, although evidence would suggest that these groups have even greater difficulties accessing benefits than the white majority population [37]. Moreover, given that the sample was drawn from people accessing the welfare rights service, the findings are derived from people more likely to be affected by financial strain and stress after cancer. Methodologically, best practice has been followed in fieldwork, data analysis and interpretation [34,36,38,39] and two individuals (EN, SM) were closely involved with data analysis and interpretation to jointly verify the findings and constantly relate interpretations back to the data. Rather than make direct generalisations from the data, we draw out the implications from our analysis and make inferences about how these findings might be transferred and applied in other settings and practices [30]. The findings of this study are likely to be of relevance to people affected by cancer in the UK, although may be less relevant to localities with less widespread and severe socio-economic deprivation. Elements of more generic relevance beyond the UK may be the added burden resulting from the financial consequences of cancer diagnosis and treatment. Relating the findings to the limited available literature backs up existing research that individuals in the UK struggle with the benefits system when left to deal with it alone [40]. Furthermore, people affected by cancer in the UK miss out on benefits to which they are entitled in the absence of specialist services [3,4] and that there are considerable additional costs associated with cancer treatment [22]. Uniquely, this study has a longitudinal element, and follow-up interviews are currently underway which will enable a more in-depth examination of the impact of the cancer trajectory on finances.

Our data demonstrate the impact that loss of earnings may have on the economic wellbeing of people with cancer and their families, as well as the additional expenditure that may accompany cancer treatment. There is a growing literature on the financial impact of cancer [5,8,41,42] largely as a response to the increasing numbers of cancer survivors, and awareness of the long term implications of loss of earnings among those of working age. Low income families were more likely to experience financial hardship than people in high income groups and within households, 'cancer in men may result in greater financial difficulties than cancer among women, although the effect will depend on breadwinner roles before diagnosis' [8] (p4350). A recent review of factors affecting cancer survivors' employment and work ability found that cancer survivors who were older, with a lower level of education and in 'blue collar' jobs were less likely to be employed following treatment [42]. The authors of this review, which did not include any UK studies, acknowledge that the likelihood of returning to work is influenced by 'the financial support that a state offers for people with cancer', as well as family resources and the psychological role of work in assisting recovery [42] (p450). The type of assistance provided by various welfare states differs greatly [9] and the findings reported here illustrate the difficulties that people with cancer in the UK face in accessing their state benefit entitlement. To the best of our knowledge, this has not been discussed elsewhere, and may be an issue that is specific to the UK welfare state system.

To date, a small body of work in the UK has shown that financial worries can add to the stress of cancer diagnosis and treatment [3,4,40]. The findings of this study demonstrate why it is difficult for those affected by cancer to pursue benefit claims themselves, but that welfare rights services transform patients' experiences and provide access to much needed benefits. In the UK, it has been shown that a significant amount of income was lost for terminally ill cancer patients [4] and lung cancer patients [3] as a result of delays before claiming, despite all patients having several contacts with a range of health and social care professionals. Given that the eligibility rules are more straightforward for people who are terminally ill, this suggests a professional failing to address holistically the full range of end of life issues. This is perhaps a manifestation of the boundaries between the NHS and social services, reflecting what are traditionally regarded as 'medical' versus 'social' issues, compounded by the complexities of the benefit system itself. Although our findings rest on a small sample, this study reinforces other work about the important role that welfare rights advice services have in facilitating access to state welfare benefits [15]. We believe that this study adds to the evidence supporting dedicated welfare rights services for people with cancer to which health and social care professionals should be able to refer. This would enable professionals to concentrate on their core business, but ensures that patients obtain the advice and assistance that they require in financial matters. Responsibility to refer would rest predominantly with health professionals, but provided adequately resourced welfare rights services are available, this could become routine practice.

The data presented indicate that patients' views differed regarding the optimal timing for referral and advice and there is clearly no 'one size fits all' remedy to this. However, given the repeated encounters that most people with cancer have with health care professionals, opportunities abound for ensuring that individuals receive information about welfare rights advice services, are able to consider it and obtain a consultation. This system avoids overburdening health care staff with non-medical responsibilities and ensures that the advice given is expert and up to date. There is a clear role for health professional involvement in ensuring that the topic of finance is raised with cancer patients as well as initiating onward referral to welfare rights advice services (in the UK, these may be provided by the local authority, Citizen's Advice Bureau or Macmillan Cancer Information Centres). Health professionals are not equipped to offer sociolegal help, unless, as in the case of Macmillan nurses, they have specialist training that is regularly updated. Other health professionals do not have the expertise to give reliable and accurate information, nor the time to do so.

The UK National Institute for Clinical Excellence (NICE) Guidance on Cancer Services recommends that 'patients and carers should be offered assistance to obtain benefits for which they are potentially eligible by professionals who are informed and knowledgeable about the benefits system' [43] (p88) and recognises that some needs of cancer patients can only be met by individuals or agencies outside the NHS. This study highlights the crucial role that welfare rights advice services have in providing such assistance. Important areas for further research include the psychological impact of dealing with the financial stresses of cancer as well as the impact of cancer on earnings and future employment prospects for those of working age.

## Conclusions

This study draws attention to the difficulties faced by people affected by cancer in the UK who do not receive their full state benefit entitlement. The findings emphasise the benefits of a welfare rights advice service that is administratively outside the NHS, but working closely with health and social care professionals who can make appropriate referrals. Such collaboration, founded on an appreciation of the full needs of people with cancer, can assist with the practical demands that arise from dealing with the illness. Without such services, many people affected by cancer in the UK will miss out on benefit entitlements at a time when they need it most, making living (and dying) with cancer more stressful than it needs to be.

## Abbreviations

**AA Attendance Allowance**: Non-means-tested benefit paid to claimants over 65 who are physically or mentally disabled and require frequent attention throughout the day and/or night in connection with their bodily function or continual supervision throughout the day and/or night. There is a lower and higher rate of the benefit. **DLA Disability Living Allowance**: Non-means-tested benefit paid to claimants under 65 who have either care or mobility needs. The care component has three rates (lower, middle and higher), while the mobility component has two rates (lower and higher). **ESA: Employment Support Allowance**: Is paid on the grounds of incapacity. There are two elements (i) contributory which is linked to national insurance contributions and (ii) income related which is means tested and can help with housing costs. It is paid to those people who have a limited capability to work due to disability or ill health. Claimants are placed in either the support group or the work related activity group, which determines the amount of ESA received. **Special Rules**: If a person is expected to live for six months or less, AA and DLA claims are prioritised, less information is required on the application form and the applicant does not have to prove how much care they need. **SSP: Statutory Sick Pay**: An earnings replacement for employees who are off work through illness. Employers must pay this to their employees who satisfy all the qualifying conditions. **Working Tax Credits**: Working Tax Credit tops up a working household's income and helps with childcare costs (although this is limited). It is means-tested and usually only paid to families on low incomes. **Job Centre**: A government-funded employment agency facility and the social security office for working-age people. Typically provides resources to enable the unemployed to find work and a system to advertise job vacancies for employers. It also provides information about training opportunities for those who have been unemployed for some time.

## Competing interests

The authors declare that they have no competing interests.

## Authors' contributions

SM initiated the study and in collaboration with Professor Martin White obtained funding. SM designed the qualitative study, EN undertook interviews and initial coding, analysis was undertaken jointly by SM, EN. Final interpretation was undertaken jointly by SM, EN and CE. SM wrote the first draft of this paper, EN and CE commented on subsequent drafts. All authors approved the final version. All authors are guarantors and accept full responsibility for the conduct of the study and the contents of this paper.

## Pre-publication history

The pre-publication history for this paper can be accessed here:

http://www.biomedcentral.com/1472-6963/10/259/prepub
